# Case reports of supernormal retrograde conduction or phase 4 block of concealed accessory pathways

**DOI:** 10.1016/j.hrcr.2025.01.011

**Published:** 2025-01-31

**Authors:** Severine Philibert, Gabriel Laurent, David Perrot, Tej Chalbia, Nicolas Lellouche

**Affiliations:** 1 Department of Cardiology, Hôpital Européen Georges-Pompidou, Paris, France; 2 Department of Cardiology, Centre Hospitalier Universitaire Dijon, Dijon, France; 3 Department of Cardiology, Hôpital Henri Mondor, Creteil, France

**Keywords:** Concealed accessory pathway, Supernormal conduction, Phase 4 retrograde conduction block, Bradycardia-dependent block, Radiofrequency ablation, Case reports

**Key Teaching Points**

• During electrophysiological studies for palpitations, dissociated retrograde conduction during baseline ventricular pacing does not rule out supernormal conduction or a bradycardia-dependent block in an accessory pathway.
• A complete scan of retrograde conduction should be systematically performed until the spontaneous orthodromic tachycardia cycle length is reached.
• Using conventional ventricular extrastimulus and/or simultaneous atrial and ventricular pacing protocols may help to identify, and sometimes differentiate, among supernormal conduction, phase 4 block, or gap phenomenon.

## Introduction

Experimental studies have demonstrated a phase of supernormal conduction in Bachmann's bundle and the His-Purkinje system.^1^, ^2^, ^3^ This supernormal conduction is associated with prolonged refractoriness in the His-Purkinje system. McHenry and colleagues^4^ reported the potential occurrence of supernormal anterograde conduction over a patent accessory pathway in 1 patient. Przybylski and colleagues^5^ suggested that this phenomenon may not be exceptional when accessory pathways exhibit an abnormally prolonged anterograde refractory period. However, retrograde supernormal conduction over accessory pathways is likely uncommon. In 1992, Suzuki and colleagues^6^ described 4 cases of retrograde supernormal conduction in concealed accessory atrioventricular pathways. We present 2 new cases of retrograde supernormal conduction over concealed accessory pathways, updating this long-known phenomenon and reaffirming the importance of thorough electrophysiological exploration.

## Case reports

### Case 1

**Figure 1:**
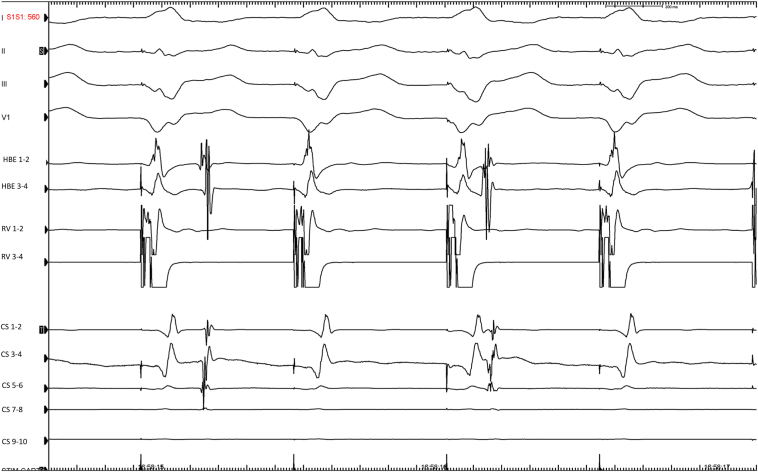
Ventricular pacing from the right ventricular apex at 600 ms down to 560 ms. A dissociated retrograde conduction can be observed at baseline and with isoproterenol. Electrocardiography leads DI-II-III and V1 are recorded along with bipolar electrograms (EGMs) from proximal coronary sinus (CS) 9–10 to distal CS 1–2, right ventricle (RV; proximal 3–4, distal 1–2), His bundle EGMs (HBE) 1–2, 3–4.

**Figure 2:**
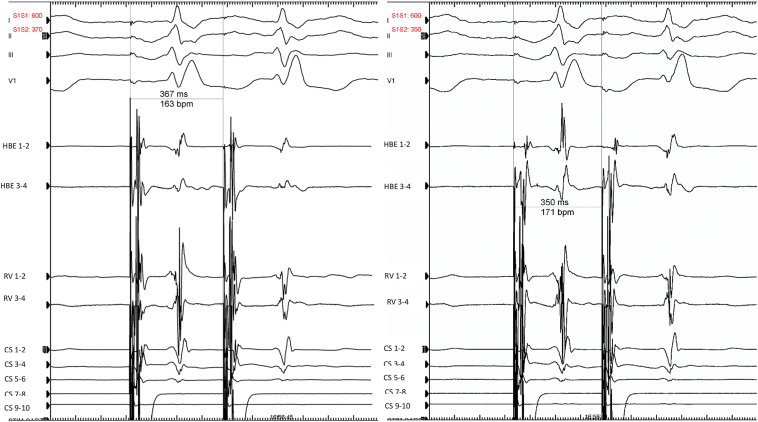
Atrial pacing extrastimulus from coronary sinus (CS) 9–10 (S1–S1: 600 ms, S1–S2: 370 down to 350 ms) showing normal atrionodal conduction with a regular right bundle branch block. HBE = His bundle electrogram; RV = right ventricle.

**Figure 3:**
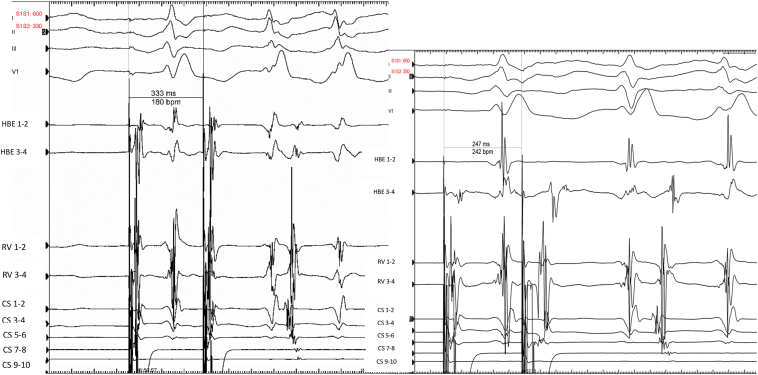
Atrial pacing extrastimulus from coronary sinus (CS) 9–10 (S1–S1: 600 ms, S1–S2: 330 ms down to 250 ms) showing normal atrionodal conduction and eccentric retrograde conduction through a left lateral accessory pathway. The earliest atrial activation is on CS 1–2, located on the lateral part of the mitral annulus (3:00 o’clock site in a left anterior oblique view). HBE = His bundle electrogram; RV = right ventricle.

**Figure 4:**
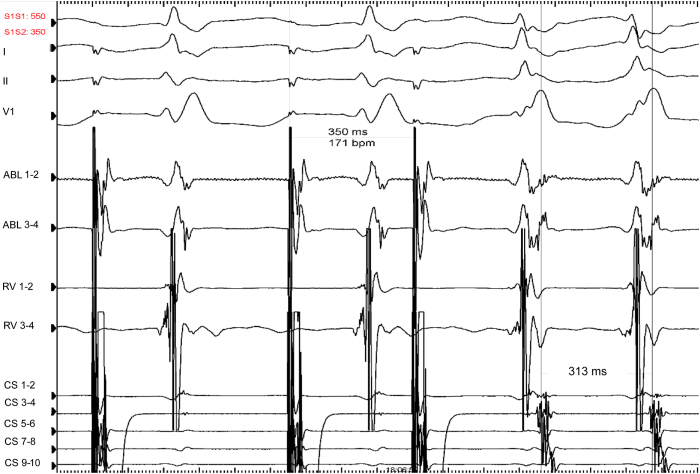
On isoproterenol, we induced an orthodromic atrioventricular reentry tachycardia at a cycle length of 313 ms with the same atrial activation sequence as before, corresponding to a concealed left lateral accessory pathway.

A 50-year-old man with no clinically significant medical history, but experiencing palpitations for many years, was admitted to our laboratory for an electrophysiological study under local anesthesia without sedation. Anterograde conduction was nodal, with no manifest preexcitation during atrial pacing at different locations. There was dissociated retrograde ventriculoatrial conduction with ventricular pacing from 600 ms to 540 ms at baseline and with isoproterenol (Figure 1). Surprisingly, we unmasked retrograde conduction through a left lateral accessory pathway while atrial pacing from the proximal coronary sinus 9–10 with a coupled extrastimulus reaching 250 ms (Figures 2 and 3). This finding was reproducible. On isoproterenol, we induced orthodromic atrioventricular reentry tachycardia (AVRT) using the retrograde left lateral accessory pathway at a cycle length of 313 ms (Figure 4).

### Case 2

**Figure 5:**
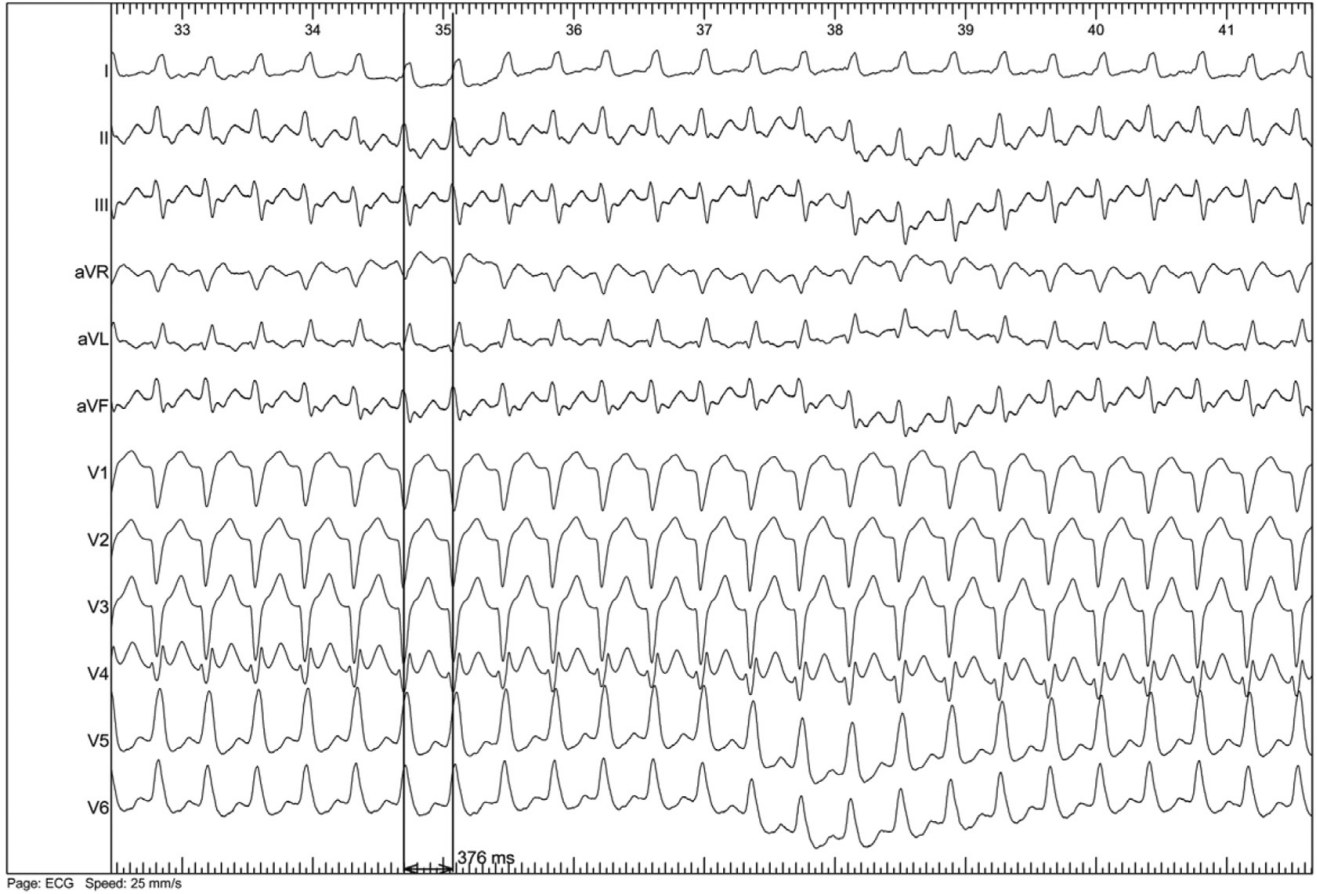
Mechanically induced atrioventricular reentry tachycardia at a stable cycle length of 376 ms with a left bundle branch block pattern.

**Figure 6:**
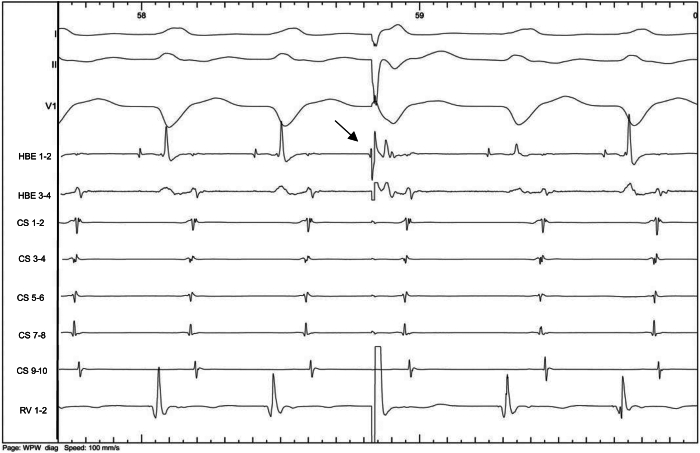
Induced orthodromic atrioventricular reentry tachycardia (AVRT) with a cycle length of 376 ms. The earliest atrial activation was at coronary sinus (CS) 7–8, located on the left posteroseptal annulus (7:00 o’clock site in a left anterior oblique view). A ventricular extrastimulus immediately after the His bundle electrogram (HBE) (*arrow*) reset the atria with the same sequence, confirming an AVRT using a concealed left posteroseptal accessory pathway. Electrocardiogram leads DI-II and V1 were recorded along with bipolar electrograms from CS leads (from proximal CS 9–10 to distal CS 1–2), right ventricle (RV) (distal 1–2), HBEs 1–2, 3–4.

**Figure 7:**
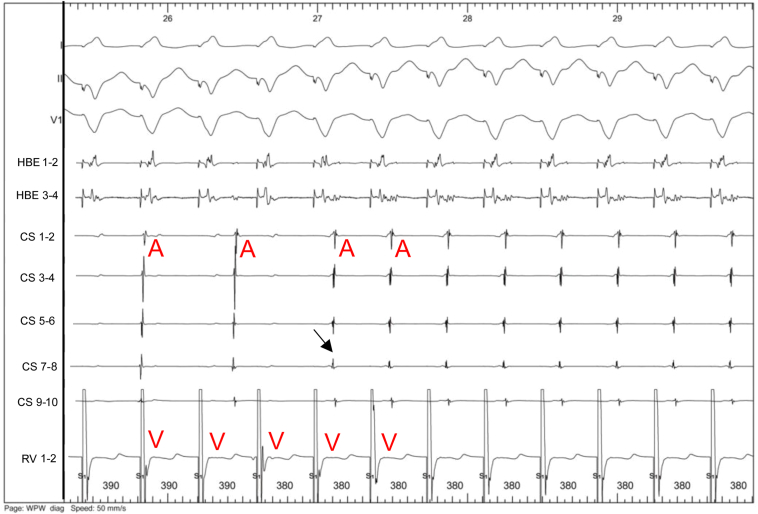
During ventricular pacing at 390 ms, we observed complete ventriculoatrial dissociation (left part of the tracing). When pacing at 380 ms (tachycardia cycle length), we unmasked 1:1 retrograde conduction with the same atrial activation sequence as previously (*arrow:* coronary sinus [CS] 7–8), corresponding to retrograde conduction through a concealed left posteroseptal accessory pathway. RV = right ventricle.

A 37-year-old patient was admitted for junctional tachycardia. The exploration was also performed under local anesthesia. Atrial pacing easily induced AVRT at a cycle length of 376 ms with a left bundle branch block pattern (Figure 5). The earliest retrograde atrial activation (coronary sinus 7–8) was reset by a ventricular extrastimulus delivered during the refractory period of the anterograde His (Figure 6). There was dissociated retrograde ventriculoatrial conduction with ventricular pacing from 600 ms to 390 ms. We then recorded 1:1 retrograde conduction through a posteroseptal accessory pathway at 380 ms, reproducing the same morphology as the clinical tachycardia (Figure 7). The ventricular overdrive pacing stopped the tachycardia without capturing the atrium.

## Discussion

Retrograde supernormal conduction or bradycardia-dependent block in the accessory pathway might be extremely rare; however, if extensive and detailed electrophysiological studies were conducted in suspected cases, the incidence might be slightly higher.

In case 1, an atrial echo occurred after premature atrial impulses only with coupling intervals from 600–330 ms to 600–250 ms at baseline. AVRT was induced on isoproterenol after a coupling interval of 600–350 ms. The absence of retrograde conduction over the accessory pathway at relatively long sinus cycle lengths at baseline (800 ms) supports the existence of a bradycardia-dependent block. Phase 4 or bradycardia-dependent conduction block may occur when cardiac tissue with automatic properties loses its resting membrane potential due to phase 4 depolarization, leaving the cell refractory to stimulation. Cases of residual retrograde phase 4 accessory pathway conduction after apparently successful radiofrequency ablation emphasized the importance of thoroughly scanning the pacing protocol, including both slow and fast rates.^7^

In case 2, we were able to induce AVRT at a cycle length of 376 ms with atrial pacing, whereas there was dissociated retrograde ventriculoatrial conduction with ventricular pacing from 600 ms to 390 ms. This supports the existence of a supernormal conduction phenomenon. However, we cannot definitively confirm this diagnosis over a phase 4 block, as we did not demonstrate retrograde conduction over the accessory pathway at relatively slow rates due to short spontaneous sinus cycle lengths. Supernormal conduction occurs due to cellular depolarization during a brief period after cellular repolarization after phase 3 of the action potential.

The 2 cases presented exhibited a very rare situation of masked concealed accessory pathways (1 left lateral and 1 left posteroseptal) due to a bradycardia-dependent block (case 1) and probable supernormal conduction (case 2). Another hypothesis was a gap phenomenon, which may occur when the effective refractory period of a distal site is longer than the functional refractory period of a proximal site. Therefore, closely coupled stimuli at the proximal site are delayed enough to allow the distal site to recover. For example, the presence of intraventricular conduction delay (delay between the spike and the ventricular electrogram) may help to understand that premature impulses may be conducted in the accessory pathway, while impulses of greater or lesser prematurity may be blocked. We have not noticed such a delay in our case 2 while ventricular pacing (Figure 7). Unfortunately, the differentiation between these phenomena could not be definitively made in our 2 cases. This distinction might have been possible by using either conventional ventricular extrastimulus or simultaneous atrial and ventricular pacing (SAVP), as described previously.^6^ The SAVP method is similar to the conventional ventricular extrastimulus testing, except that the basic drive consists of a series of SAVP. The SAVP method from the high right atrium or the coronary sinus may either reveal (while not seen with a ventricular extrastimulus pacing protocol) or modify the range of supernormal conduction over an accessory pathway (compared with right ventricular pacing only).^6^

Different atrial pacing sites may produce different results (high right atrium vs coronary sinus). Although the exact mechanism explaining this difference is unknown, it may be related to the fact that the improvement of refractoriness of the accessory pathway can be greater when the coronary sinus is simultaneously paced than when the high right atrium is. This method may even help to reveal a gap phenomenon either dependent on a slow conduction over the accessory pathway itself or due to the presence of intraventricular conduction delays (delay between the spike and the ventricular electrogram). As the procedures were performed under local anesthesia, we can rule out involvement of vagal hyperactivation due to sedation.

## Disclosures

The authors have no conflicts of interest to disclose.
